# Chitosan Rate of Uptake in HEK293 Cells is Influenced by Soluble *versus* Microparticle State and Enhanced by Serum-Induced Cell Metabolism and Lactate-Based Media Acidification

**DOI:** 10.3390/molecules18011015

**Published:** 2013-01-15

**Authors:** Caroline D. Hoemann, Jessica Guzmán-Morales, Nicolas Tran-Khanh, Geneviève Lavallée, Mario Jolicoeur, Marc Lavertu

**Affiliations:** Department of Chemical Engineering, Institute of Biomedical Engineering, FRQS Groupe de Recherche en Sciences et Technologies Biomédicales (GRSTB), Ecole Polytechnique, 2900 Blvd. Edouard Montpetit, Montreal, Quebec, H3T 1J4, Canada; E-Mails: jessica.guzman-morales@polymtl.ca (J.G.-M.); nicolas.tran-khanh@polymtl.ca (N.T.-K.); gennygenlavallee@hotmail.com (G.L.); mario.jolicoeur@polymtl.ca (M.J.); marc.lavertu@polymtl.ca (M.L.)

**Keywords:** chitosan, HEK293 cells, lactate, EGF, serum, confocal microscopy, microparticle

## Abstract

Chitosan is a biocompatible polysaccharide composed of glucosamine and *N*-acetylglucosamine. The polymer has a unique behavior of fluctuating between soluble chains at pH 6 and insoluble microparticles at pH 7. The purpose of this study was to test the hypothesis that chitosan structure, solubility state, and serum influence the rate of cell uptake. Chitosans with 80% and 95% degree of deacetylation (medium and low viscosity) were tagged with rhodamine and analyzed for particle size, media solubility, and uptake by HEK293 epithelial cells using live confocal microscopy and flow cytometry. In media pH 7.4 with or without 10% serum, chitosans fully precipitated into 0.5 to 1.4 µm diameter microparticles with a slight negative charge. During 24 h of culture in serum-free medium, chitosan particles remained extracellular. In cultures with serum, particles were taken up into intracellular vesicles in a serum dose-dependent manner. Opsonization of chitosan with serum, or replacement of serum by epidermal growth factor (EGF) failed to mediate serum-free chitosan particle uptake. Serum stimulated cells to acidify the media, partly by lactate generation. Media acidified to pH 6.5 by 7 mM lactate maintained 50% of chitosan in the soluble fraction, and led to minor uniform serum-free uptake in small vesicles. Conclusion: Media acidification mediates minor *in vitro* uptake of non-biofouled soluble chitosan chains, while serum-biofouled insoluble chitosan microparticles require sustained serum exposure to generate energy required for macropinocytosis.

## 1. Introduction

**Figure 1:**
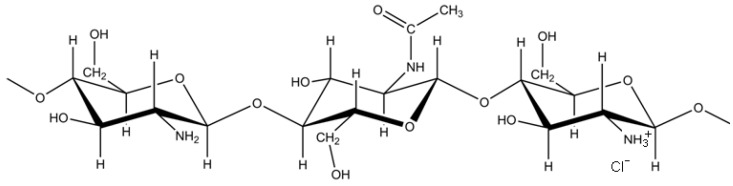
Chemical structure of chitosan, a polysaccharide with heterogeneous GlcNA content.

Chitosan (Figure 1) is a mucoadhesive polysaccharide with wide-ranging applications in drug delivery, gene delivery, vaccines, and tissue engineering [1,2]. The mode of delivery, site of action, biodegradation, and cell/tissue responses to chitosan are tightly linked to the polymer structure. Chitosan is derived from chitin, β(1-4)-*O*-linked *N*-acetyl-β-D-glucosamine (GlcNA), the insoluble backbone component of shrimp and crab shells, by chemical deacetylation. Depending on the degree of deacetylation (DDA), chitosan can have an average glucosamine content that ranges from ~45% to 100% (Figure 1). Chitosan is soluble at pH 6, where glucosamine carries a minimal ~40% positive charge state [3], and insoluble at pH 7, except for chitosans with very low DDA (~50% DDA) [4] or very low molecular weight (chitosan oligomers, <3,000 Da) [5,6].

Enzymatic degradation of chitosan by serum lysozyme requires three consecutive GlcNA residues to hydrolyze the sugar linkages [7]. Highly deacetylated chitosans (>90% DDA) are non-biodegradable [8] and slightly cytotoxic [9], while more acetylated chitosans (50% to 85% DDA) are biodegradable, and attract neutrophils and macrophages which phagocytose and degrade the polymer [8,10,11,12,13]. After becoming hydrolyzed to ultra-low molecular weight (<10,000 Da), chitosan chains pass across the intestinal epithelial barrier [14] and become excreted in urine [4]. In the context of drug delivery, chitosan is often used as a nanoparticle [9,15] or a soluble PEGylated form [16]. In the context of regenerative medicine and vaccination adjuvants, chitosan is often applied or implanted as a slightly acidic solution [17,18], microparticles [19], or insoluble scaffolds [8].

Our specific interest in studying chitosan-cell interactions occurs in the context of regenerative medicine, where therapeutic effects on bone and cartilage repair were obtained using an implant composed of blood clot interspersed with insoluble chitosan microparticles (80% DDA, medium viscosity) [20]. The bio-engineered chitosan-blood clot can be injected and coagulated *in situ* in a surgically treated cartilage lesion, using medium viscosity chitosan solutions whereas low-viscosity chitosan solutions generate runny blood mixtures that can only be implanted after pre-solidifying *ex vivo* [21]. Others have used viscous acetic acid chitosan solutions applied to bleeding tissues as a hemostatic, which is predicted to generate insoluble microparticles at blood pH [17]. Chitosan-based gene therapy also requires co-delivery of insoluble chitosan microparticles [22,23,24]. Examples of biomedical uses of chitosan as insoluble microparticles are summarized in Table 1.

**Figure 2:**
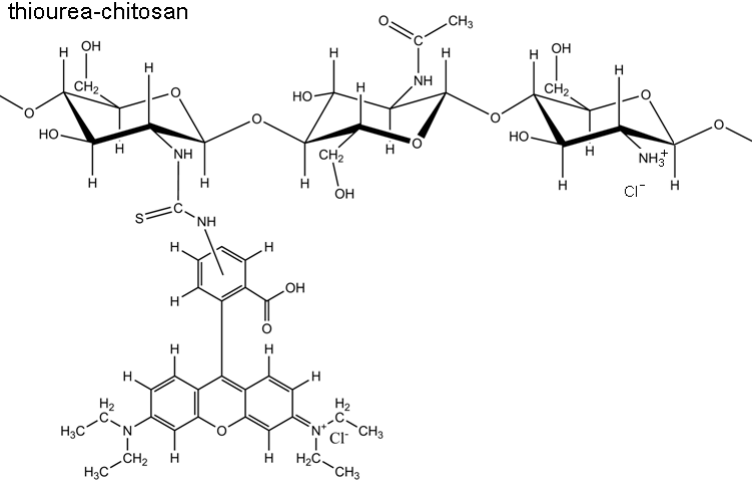
Chemical structure of RITC-chitosan.

Little information is available on conditions that control chitosan microparticle uptake by non-phagocytic cells, however several *in vitro* studies indicate a role for serum. Fluorescent chitosans with broad structural characteristics (46% to 92% DDA, 10–213 kDa) showed negligible uptake in serum-free media, by several epithelial cell types [9,15,32,33], even when media was adjusted to pH 6.5 below the intrinsic p*K*_a_ (p*K*_0_ ~ 6.7) of chitosan [3], to favor polymer solubility via positive charge state. But these studies used chitosan tagged with fluorescein, which is quenched at acidic pH, and live cell chitosan uptake could not be directly monitored or quantified. In other studies using rhodamine-labeled chitosan, an acid-resistant fluorophore (Figure 2) [34], a variety of cell types internalized chitosan in serum-containing media: primary bone marrow stromal cells, neutrophils, bone marrow monocyte-derived macrophages, and HEK293 cells [13,22,26,27,28]. One study suggested that 92% DDA, 10 kDa chitosan can be internalized by HEK293 cells with or without serum, but the conclusions were only based on flow cytometry data and for only one particular chitosan [26]. A systematic study of the effect of chitosan structure on microparticle uptake by cells, with or without serum, is currently lacking.

Exposure of a biomaterial to serum will lead to “biofouling”, or natural adsorption or deposition of biological factors from the environment onto the biomaterial surface. Cationic chitosan chains were previously shown to be selectively “biofouled” with anionic serum proteins, including highly abundant serum albumin (~40 mg/mL in serum, pI 5.7) [35] and complement C3 (~1 mg/mL in serum, pI 6.0) [36]. Complex formation with C3 and other serum factors has an unclear influence on chitosan-cell interactions. It is well-established that ≤200 nm diameter chitosan nanoparticles (chitosan condensed into polyplexes with DNA or polyphosphate) are readily internalized with and without serum, especially at acidic pH [9,26,37]. The purpose of this study was to systematically analyze the effect of chitosan structure on chitosan microparticle formation and uptake in live cells, using HEK293 cells, a transformed epithelial human cell line with a high endocytotic capacity. Four structurally distinct chitosans were specifically labeled at 1 rhodamine tag per 200 monomers (*i.e.*, 0.5% mol rhodamine isothiocyanate, RITC/mol chitosan) [33], which minimally alters DDA level and molecular weight, and allows direct, quantitative comparison by flow cytometry. We tested the hypothesis that *N*-acetyl- glucosamine (GlcNA) content and serum influence the rate of cell uptake.

## 2. Results and Discussion

### 2.1. Structural Characterization of RITC-Chitosan and RITC-Chitosan Microparticles

**Figure 3:**
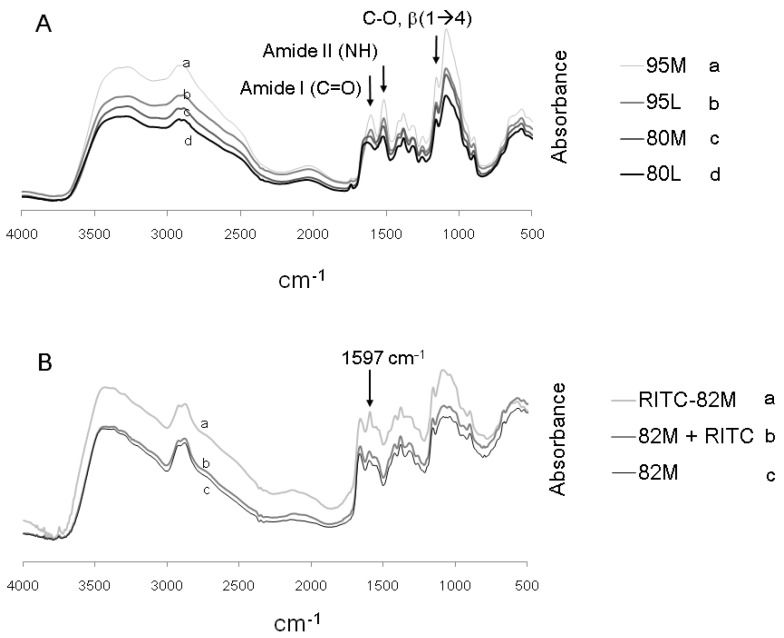
FT-IR spectra of the chitosan library prior to RITC coupling (**A**), and RITC-82M chitosan *vs.* 82M chitosan-only and 82M chitosan mixed with RITC without coupling (**B**). Chitosans were analyzed in the HCl salt conjugate form (**A**) or free base form (**B**). 95: 95%DDA; 80: 80%DDA; 82: 82%DDA; M: medium viscosity; L: low viscosity.

Four chitosans were used to generate a library of fluorescent derivatives for cell-uptake studies, at 95% DDA or 80% DDA and medium or low viscosity, while another 82% DDA chitosan was used for structural analyses before and after rhodamine tagging (Table 2). Photoacoustic Fourier Transform Infrared (FT-IR) spectroscopy confirmed structural differences in the library of four chitosans prior to derivatization (Figure 3A), and demonstrated covalent linkage of RITC to 82M chitosan (Figure 3B).

By FT-IR, chitosans 80M and 80L had a diminished amide II peak (NH bending in Glc, 1561 cm^−1^) and a broader amide I peak (C=O carbonyl stretching in GlcNA, ~1640 cm^−1^) compared to 95M and 95L (Figure 3A). The amide I peak can appear as a doublet at 1663 cm^−1^ and 1626 cm^−1^ when 2 types of intermolecular H-bonding are involved with the carbonyl group [38]. Higher molecular weight chitosan (95M *vs.* 95L, and 80M *vs.* 80L) showed relative intensification of the β(1-4) *O*-linkage peak at 1159 cm^−1^ (C–O stretch) [38,39] (Figure 3A). The strong 1597 cm^−1^ peak in RITC-82M chitosan (arrow, Figure 3B) is consistent with a new thiourea linkage between RITC and the amine group of the glucosamine monomer.

**Figure 4:**
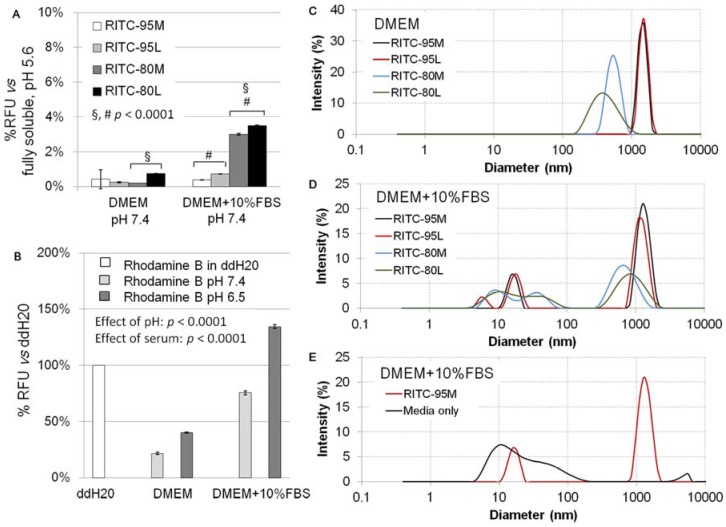
Residual neutral-soluble RITC-chitosan fluorescence in media pH 7.4 *vs.* RITC-chitosan fluorescence in water pH 5.6 defined as 100% (**A**), % rhodamine B fluorescence in culture media *vs.* in water defined as 100% (**B**), and hydrodynamic chitosan particle size distribution in DMEM pH 7.4 (**C**) and DMEM+10% FBS pH 7.4 (**D**); Panel **E** shows 95M in DMEM+10% FBS (red line) compared to media-alone (DMEM+10% FBS, black line).

**Figure 5:**
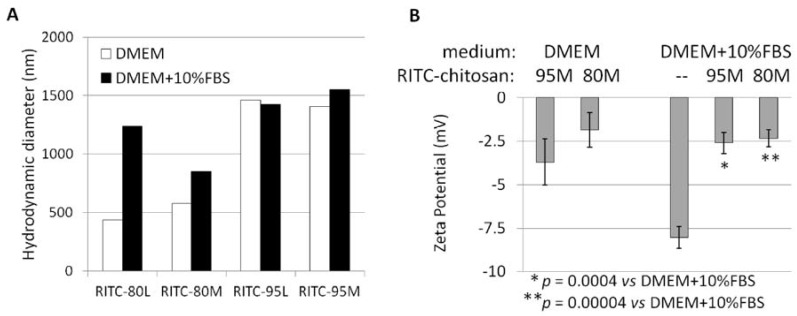
Average hydrodynamic diameter of the chitosan library with and without serum (**A**) and zeta potential of 50 µg/mL RITC-95M or RITC-80M chitosan in media without and with 10% serum (N = 3 to 4 measures, mean ± standard deviation, **B**).

RITC-chitosans pipetted into serum-free medium pH 7.4 rapidly formed >99% insoluble microparticles (DMEM, Figure 4A), with an average hydrodynamic diameter of ~0.5 µm (80M, 80L) or ~1.4 µm (95M, 95L, Figure 4C and Figure 5A), and a slight negative charge (−2 to −3.7 mV, Figure 5B). RITC-chitosans pipetted into medium with 10% serum pH 7.4 also precipitated, with a slight but significant ~3% increase in neutral solubility for 80M and 80L but not 95M and 95L (*p* < 0.0001, N = 6, Figure 4A). Rhodamine B fluorescence was maintained in all cell culture media, but depressed in media pH 7.4 compared to water, and enhanced by serum and acid pH (Figure 4B). In medium with 10% serum, RITC-chitosan formed polydisperse electronegative (−2.5 mV) microparticles with average diameters of ~1.0 µm (80M, 80L) and ~1.5 µm (95M, 95L), along with new nanoparticle peaks (Figure 4D and Figure 5).

Media-alone with 10% serum contained particles ranging from 7 to 100 nm (black line, Figure 4E) with a negative zeta potential (−8 mV, Figure 5B). These data are compatible with the hydrodynamic diameter (7 nm) of BSA and anionic charge state at neutral pH [40]. Note that BSA is present at around 80-fold excess over RITC-chitosan in the samples with 10% serum and 50 µg/mL chitosan. Addition of chitosan to media with 10% serum reproducibly reversed the electronegative zeta potential reading (Figure 5B).

To summarize, dynamic light scattering (DLS) measures show that chitosan formed 0.2 µm to 2 µm insoluble microparticles and microparticle aggregates at pH 7.4, with and without serum. In serum-free medium, chitosan microparticles are potentially complexed with anionic media salts or other anionic components (amino acids). In culture medium with serum and chitosan, small particles (<100 nm) represent mainly serum proteins, a fraction of which are most probably complexed with individual chitosan chains along with larger chitosan microparticle aggregates (>200 nm) that are expected to be partly complexed with anionic serum factors [35,36].

### 2.2. Serum Stimulates RITC-Chitosan Microparticle Uptake

**Figure 6:**
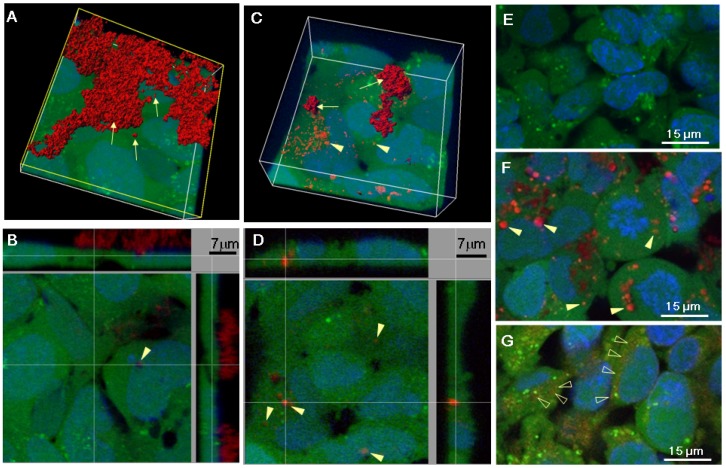
Confocal images of HEK293 cells cultured for 24 h with 80M RITC-chitosan in DMEM pH 7.4 (**A**–**B**) or DMEM 10% FBS pH 7.4 (**C**–**D**) (3-D reconstructions). Panels **E**–**G** show HEK293 cells in media with 10% FBS (**E**) incubated 24 h with 95M RITC-chitosan (**F**) or rhodamine B (**G**). After chitosan uptake, cells were labeled with calcein AM (green live cell cytosol) and Hoechst 33342 (blue cell nuclei). Arrowheads: intracellular chitosan; arrows: extracellular chitosan. Open arrowheads: rhodamine B.

Live confocal microscopy showed that in DMEM-only, RITC-chitosan particles collected on the cell surface without being internalized after 24 h of culture (Figure 6A–B). Only rare vesicular RITC-chitosan uptake was seen in DMEM pH 7.4 (arrowhead, Figure 6B). In medium with 10% FBS, all 4 RITC-chitosans became readily internalized in 0.1 to 2 µm diameter vesicles (arrowheads, example images of RITC-80M Figure 6C-D and RITC-95M, Figure 6F). Cells exposed to free rhodamine B showed a faint staining pattern (Figure 6G *vs.* Figure 6E), similar to the mitochondrial stain previously reported for porcine kidney proximal tubule cells exposed to 0.1 µg/mL rhodamine B in serum-containing medium [41]. Because RITC-chitosan was detected in intracellular vesicles while free rhodamine B fluorophore became associated with structures resembling mitochondria, this showed that the fluorescent signal observed inside cells was intact RITC-chitosan and not hydrolyzed fluorophore.

**Figure 7:**
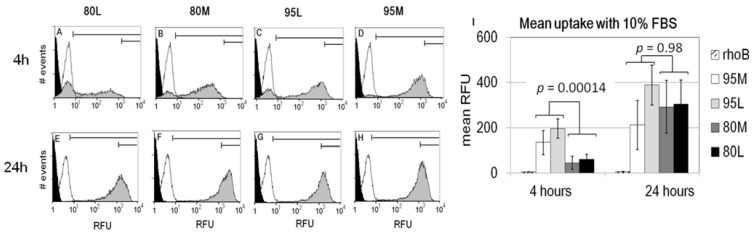
Flow cytometry analysis of rhodamine B and RITC-chitosan uptake after 4 h (**A**–**D**) or 24 h (**E**–**H**) of culture in 10% FBS, for 4 structurally distinct chitosans (as indicated), and mean RFU values from N = 3 to 5 distinct experiments (**I**). For **A**–**H**: Black peak: unlabeled cells; white peak: rhoB-labeled; grey peak: RITC-chitosan-labeled.

When analyzed by flow cytometry, cells incorporated more 95% DDA than 80% DDA chitosan after 4 h of culture with 10% serum (*p* = 0.00014, Figure 7A–D,I). Similar DDA-dependent uptake was previously reported for chitosan-polyphosphate nanoparticles in A549 cells after 4 h in a serum-free balanced salt solution [9]. However the data could have an artifact because in our experiments, highly insoluble 95M and 95L chitosan particles were observed to settle more rapidly to the bottom of the petri. After 24 h of culture in 10% serum, HEK293 cells internalized all four RITC-chitosans to the same high level (Figure 7E–I). Free rhodamine B fluorophore was rapidly incorporated in HEK293 cells with low and steady fluorescence at 4 and 24 h (white peak, Figure 7). Rhodamine B was added to the cell cultures at the same molar level as the RITC fluorophore in our chitosan derivatives. Therefore, the ~200-fold higher fluorescence in RITC-chitosan-fed cultures (Figure 7I) reflects uptake of more fluorophore attached to chitosan. Part of the higher RITC-chitosan fluorescence at 24 h could be due to acid-induced intensification of rhodamine B fluorescence in acidifying endosomes (Figure 4B).

**Figure 8:**
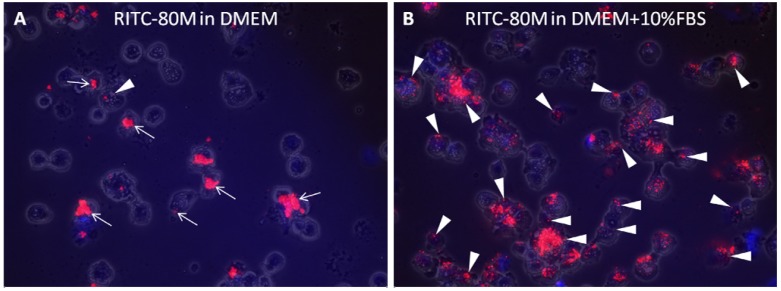
Merged epifluorescent-brightfield images of cytospins of HEK293 cells submitted to flow cytometry after culturing 24 hours with RITC-80M in DMEM (**A**) or in DMEM+10% FBS pH 7.4 (**B**). Red signal is RITC-chitosan, blue is Hoechst 33346-stained DNA. Symbols: arrows: extracellular RITC-80M adsorbed to cell surfaces as shown by the non-uniform microparticle shape; arrowhead: intracellular vesicles with RITC-chitosan.

Cytospins of cells submitted to flow cytometry analysis showed that some of the signal for cells cultured in serum-free medium arose from RITC-chitosan adsorbed to the cell surface (arrows, Figure 8A). The contribution of extracellular signal was unavoidable, as trypan blue which is often used to mask extracellular fluorescein green fluorescence emission [32,42], is unable to mask extracellular rhodamine emission [26]. By comparison, cytospins of cells cultured with RITC-chitosan in 10% serum showed abundant fluorescent intracellular vesicles (Figure 8B). These data allow us to conclude that high cell fluorescence after 24 h of culture is due to intracellular accumulation and not to more adsorption of RITC-chitosan to the cell surface. Taken altogether, these data confirm the hypothesis that DDA influences the rate of cell uptake (Figure 7I), but show that the delayed uptake of 80%DDA is a technical issue related to time required for the microparticles to collect on the monolayers.

### 2.3. Role of Serum and Lactate in Mediating Chitosan Uptake by HEK293 Cells

**Figure 9:**
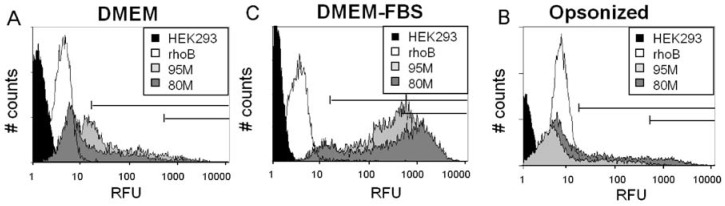
Flow cytometry profiles of cells exposed for 24 h to RITC-chitosan microparticles in DMEM (**A**), in DMEM+10% FBS (**B**), or FBS-opsonized RITC-chitosan microparticles in DMEM (**C**). All media was pH 7.4. White peak: free rhodamine B; black peak: unlabeled HEK293 cells; light grey peak: RITC-95M; dark grey peak: RITC-80M.

Our data indicated that serum was necessary for chitosan uptake but did not fully explain why. It was previously shown that anionic serum proteins such as complement C3 associate selectively and non-covalently with chitosan, through electrostatic interactions [36]. We therefore tested the hypothesis that opsonization of chitosan microparticles by serum proteins is sufficient to promote chitosan microparticle uptake. However serum-opsonized chitosan particles were not internalized by HEK293 cells after 24 hours in serum-free media (Figure 9).

**Figure 10:**
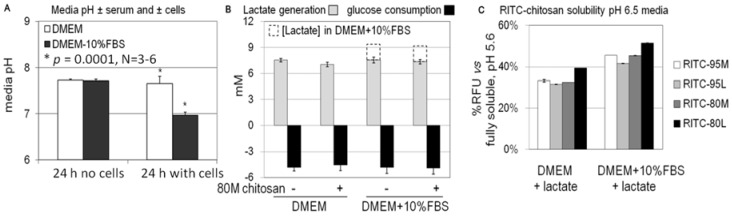
Media pH (**A**), and corresponding 7 mM lactate generation and 5 mM glucose consumption after 24 h of incubation at 37 °C with and without serum (**B**). RITC-chitosan added to lactate-acidified media pH 6.5 remained 30% to 50% soluble compared to RITC-chitosan fully soluble in dilute HCl pH 5.6 (**C**).

Confluent monolayers were observed to acidify the media after 24 h specifically in the presence of serum, from an initial pH ~7.6 to final pH ~7.0 (Figure 10A). These data suggested that serum was stimulating cells to produce more lactate. However after 24 h of culture, HEK293 cells generated the same levels of lactate (~7 mM per million cells) and consumed a similar level of glucose (5 mM) with and without serum (Figure 10B). The final lactate concentration was slightly higher in serum-containing conditioned media due to the presence of 1.9 mM lactate in DMEM+10% serum (dashed bar, Figure 10B). Exposure of cells to chitosan had no influence on glucose consumption or lactate generation (Figure 10B).

In other experiments, it was determined that addition of 7 mM lactate to cell culture media (*i.e.*, the same amount of lactate produced during 24 h by monolayer cells) reproducibly acidified the media by approximately 1 pH unit, with or without 10% serum. The data seem to present a contradiction because 7 mM lactate produced by cells cultured in DMEM for 24 h fails to generate a drop in media pH. It should be noted however that the cell cultures are maintained under 5% CO_2_ in media with a bicarbonate buffering system that helps neutralize the conditioned media pH and off-set the lactate production in DMEM.

To determine whether lactate could be directly influencing chitosan solubility and uptake, RITC-chitosan was pipetted into fresh lactate-acidified media pH 6.5. None of the chitosans fully precipitated, remaining 30% to 50% soluble at pH 6.5, with or without serum (Figure 10C). The solubility data shown in Figure 10C are consistent with the chitosan intrinsic p*K*_a_, which predicts around 50% solubility at pH 6.5 in isotonic solutions [3].

**Figure 11:**
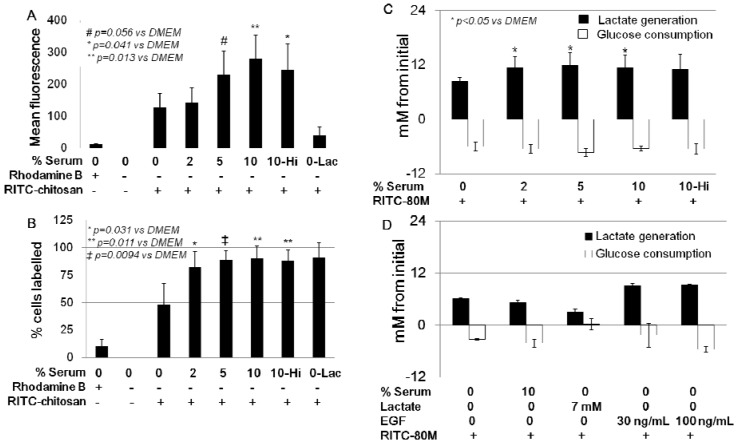
Quantitative flow cytometry analysis of HEK293 cells (**A**–**B**, mean fluorescence and % cells labeled), and lactate generation (**C**–**D**) after 24 h of culture with RITC-80M chitosan in media with different levels of serum (0%, 2%, 5%, 10%), exogenous lactate or EGF. 10-Hi: 10% heat-inactivated serum; 0-Lac, DMEM+7 mM lactate.

When cells were exposed for 24 h to RITC-chitosan in lactate-acidified DMEM pH 6.5, according to quantitative flow cytometry, nearly all cells were labeled, but at very low levels (0-Lac, Figure 11A–B). When cells were exposed to RITC-chitosan in media containing 2% to 10% serum, a serum dose-dependent uptake was seen (*p* < 0.05, Figure 11A–B). Heat-inactivated FBS, which is depleted of complement activity and a number of important chemokines and growth factors [43,44], slightly suppressed mean cell fluorescence after 24 h of uptake (RITC-80M), without influencing the percent cells labeled (Figure 11A–B). All of the cultures carried out with 2% to 10% serum at pH 7.4 showed the same glucose consumption and slightly higher lactate generation than DMEM-only (Figure 11C). Lactate generation, however, did not correlate with chitosan uptake levels (Figure 11C *vs.* Figure 11A).

**Figure 12:**
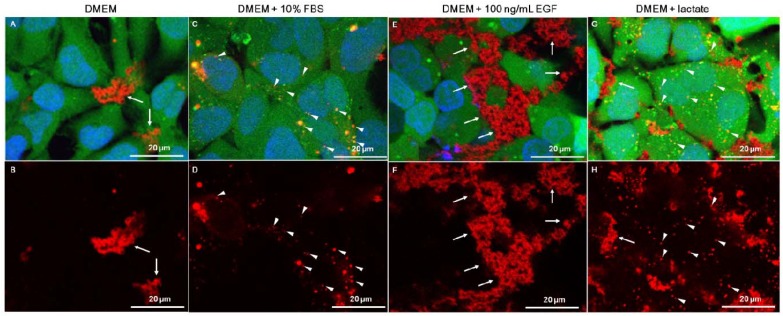
Confocal images of HEK293 cells exposed to RITC-80M chitosan pipetted into media pH 7.4 (**A**–**F**) or lactate-acidified media pH 6.5 (**G**–**H**). Top panels: calcein AM (green live cells), Hoechst 33342 (blue cell nuclei) and RITC-80M chitosan particles (extracellular, white arrow; vesicle, arrowhead). Bottom panels: RITC-chitosan-only. Arrows: extracellular chitosan. Arrowheads: intracellular chitosan.

We next tested whether a heat-sensitive serum factor, such as epidermal growth factor (EGF) which stimulates Rac1-dependent macropinocytosis [45,46], could be sufficient to mediate chitosan microparticle uptake in serum-free media. Cells stimulated with 30 ng/mL or 100 ng/mL EGF in serum-free media pH 7.4 showed increased lactate production (9 mM with EGF *vs.* ~6 mM for DMEM-only) and higher glucose consumption (5.3 mM with EGF *vs.* 3.3 mM for DMEM-only, Figure 11D) but failed to internalize chitosan (Figure 12E–F). In other cultures, we tested the hypothesis that lactate alone can stimulate serum-free chitosan uptake. Exogenous lactate suppressed lactate generation (3.1 mM *vs.* 6 mM for DMEM-only) and glucose consumption (0.2 mM *vs.* 3.3 mM, Figure 11D), and promoted RITC-chitosan uptake in very small vesicles in nearly all of the cells (Figure 12G–H).

This study reports novel data showing that ≥2% serum promotes significant uptake of biofouled biodegradable and non-biodegradable insoluble chitosan microparticles in HEK293 cells, after enough time has elapsed to allow the particles to fully settle on the cell monolayer. RITC-chitosan particles and many intracellular vesicles had a similar diameter, suggesting that each vesicle could contain 1 or a few RITC-chitosan microparticles. This notion, together with the FACS analysis performed on cells incubated in graded serum concentrations, suggests that higher levels of serum promote cells to take up more individual particles per cell—rather than more chitosan particles per vesicle.

High-glucose media (DMEM, DMEM with serum and DMEM with EGF) all stimulated cell glycolysis, but only serum promoted significant media acidification and insoluble RITC-chitosan particle uptake. These data are consistent with a report that serum specifically stimulates mitochondrial oxidative phosphorylation and CO_2_ generation [47], which is acidifying through the bicarbonate buffer system (Figure 10A). These collective data suggest that serum most probably drives higher cell energetics required for membrane ruffling in macropinocytosis [48]. It is also possible that serum-stimulated cell metabolism helps the cell generate local acid gradients at the cell membrane that dissociate chitosan aggregates, or confer slight positive charge to particles and favor interaction of single chains with the cell membrane, and thereby facilitate uptake of membrane-bound chitosan. Biofouling of chitosan particles with anionic serum proteins could also potentially facilitate break-up of chitosan aggregates. Once internalized, as for any other macromolecular nutrient, chitosan provides the cell with an organic energy source, but may take a while to become metabolized as the label persists in intracellular vesicles for several weeks and cell passages (unpublished observations).

Particle size and cell adhesion both play a role on biomaterial uptake in serum-free media. Smaller nanoparticles of chitosan-DNA, chitosan-polyphosphate, and even serum-coated polystyrene are internalized by non-phagocytic cells under serum-free conditions [9,15,25,26,33,49]. HeLa epithelial cells show only minor uptake of individual 0.5 to 3 µm diameter spherical particles after 4 hours in serum-free media, although higher uptake is seen for particles carrying a positive surface charge (amino-methacrylate-containing hydrogel particles, polyethylinimine-coated latex beads) [16,42]. Cells may be unable to internalize chitosan microparticles in serum-free media pH 7.4 partly because the slightly electronegative salt particles have a poor capacity to bind to the cell membrane (Figure 6B). In addition, at pH 7.4 chitosan forms high molecular weight aggregates (Figure 6A and Figure 12E), too large to be internalized by non-phagocytic cells. Soluble individual macromolecules such as hyaluronan (negative charge) [50], dextran (neutral), and lactate acid-soluble chitosan (positive charge, Figure 12G–H) could enter the cell more easily by fluid-phase pinocytosis. Note that the effective concentration of chitosan at the cell surface is diminished in acidified media where half of the chitosan is in the fluid-phase, compared to media at neutral pH that generates 99% insoluble chitosan particles that fully collect on the monolayer surface. Tissue acidification to pH 6.5 required for uptake of soluble cationic chitosan chains may be present only in special circumstances *in vivo*, in cases of hypoxia in healing wounds, or rapidly growing tumors [51].

Data generated by this study show that the volume of culture media controls the rate of uptake, because it takes longer for chitosan particles to settle in a thick layer of media. These results suggest new methods for promoting rapid chitosan particle uptake *in vitro*, for example, by pipetting insoluble microparticles over cells with only a thin layer of serum-containing media, or by collecting particles and cells into a loose pellet to maximize particle-cell contact. These data also suggest that the pH of the chitosan delivery system could have an important influence on chitosan dispersion *in vivo* at physiologic pH.

Translation of chitosan for *in vivo* applications needs to place *in vitro* culture systems into perspective. Serum-stimulation of *in vitro* cultured cells simulates a context of wounding, where bleeding, coagulation, and edema promoting platelet degranulation are present. Serum drives chitosan uptake *in vitro* by mesenchymal stem cells and epithelial cells [22,27,52], and in this study, 95% DDA chitosan was internalized more rapidly *in vitro* by HEK293 cells than 80%DDA chitosan (Figure 7I). However*,* when implanted *in vivo* as microparticles formed inside a blood clot, 80% DDA chitosan is more rapidly taken up by cells than 95% DDA chitosan [53], namely by neutrophils and macrophages [11,12,21,54]. Targeted delivery of chitosan microparticles to non-phagocyte cells *in vivo* may require the use of non-biodegradable chitosan or chitosan derivatives to elude innate immune cell clearance [24]. Future design of chitosan particles that guide specific *in vivo* responses will require a better understanding of innate immune reactions to insoluble chitosan microparticles.

## 3. Experimental

### 3.1. Materials

Medical-grade chitosans (80.6% DDA, 81.5% DDA, 94.5% DDA, <0.2% protein, <500 Endotoxin Units (EU)/g) were provided by BioSyntech (now Piramal Healthcare Canada, Laval, QC, Canada). RITC was covalently coupled to chitosan at 0.5% mol RITC/mol chitosan (Table 1) or 1.0% mol/mol (81.5% DDA) and the molecular weight determined as previously described [34]. HEK293 cells were purchased from the American Type Culture Collection (Catalog N° CRL-1573, Manassas, VA, USA) and used between passage 7 and 28. Hoechst 33342, calcein AM, Dulbecco’s Modified Eagle Medium with High Glucose (DMEM-H, with 25 mM D-glucose) (Gibco Product N°, 12100-046, with 2.2 g/L sodium bicarbonate), and trypsin-ethylenediaminetetraacetic acid (trypsin-EDTA), and recombinant epidermal growth factor (EGF) were from Invitrogen (Mississauga, ON, Canada). Costar 6-well plates and 30 mm tissue culture petris, Corning 0.22 µm syringe filters, and 96-well black assay FluoroNunc flat-bottom plates, were from Fisher Scientific (Montreal, Canada). Fetal Bovine Serum (FBS), rhodamine B, Dulbecco’s phosphate buffered saline (PBS), L-(+)-Lactic acid solution (90% purity), and 1 N hydrochloric acid (cell culture grade) were from Sigma-Aldrich (Oakville, ON, Canada).

### 3.2. Photoacoustic Fourrier Transform Infrared Spectroscopy (PAS FT-IR)

Spectral analyses were carried out on chitosans with either medium viscosity (80M, 82M, 95M) or low viscosity (80L, 95L) prior to RITC derivatization or RITC-82M (Table 1). Samples 80L, 80M, 95L and 95M were freeze-dried as an HCl salt conjugate while 82M chitosan and RITC-chitosan 82M were alkaline-precipitated, rinsed with deionized water to neutral pH, then freeze-dried in free base form. Samples were stored in dessicant prior to PAS FT-IR analysis of dry powder under helium gas with a Digilab FTS6000 FT-IR spectrometer equipped with a Photoacoustic cell (MTEC Model 300 Detector, MTEC Photoacoustics, Inc., Ames, IA, USA). Samples and the background scan were performed at room temperature with a spectral resolution of 8 cm^−1^ using the rapid scan mode at 2.5 KHz.

### 3.3. Particle Size and Zeta Potential

A Malvern particle sizer (Nano-AS, Malvern Instruments, Worcestershire, UK) was used to determine RITC-chitosan particle size and zeta potential in DMEM and DMEM+10% FBS. RITC-chitosan particles were formed by pipetting 10 µL of 5 mg/mL RITC-chitosan into 1 mL of culture medium pH 7.4 with gentle mixing, and particle size or zeta potential was measured after 5 minutes of incubation at room temperature in a closed cryovial. Size data from intensity weighted distribution were calculated as the average of 2 independent reads of the major peak. Zeta potential is reported as the mean ± standard deviation obtained from duplicate measures of 2 independent samples prepared on separate occasions. Measures of DMEM-only showed negligible particle levels in the medium.

### 3.4. Measurement of RITC-Chitosan Solubility in Cell Culture Media

RITC-chitosan was dissolved at 5 mg/mL in dilute HCl (20.7 mM HCl for 80% DDA chitosan and 24.3 mM HCl for 95% DDA chitosan) to produce pH 5.6 solutions with 90% degree of protonation of free amine groups. These solutions were 0.22 µm filter-sterilized with no loss of fluorescence and stored flash-frozen at −80 °C as aliquots that were freeze-thawed a maximum of 3 times, as additional freeze-thaw was observed to depress fluorescence. For solubility tests, RITC-chitosan was diluted to 50 µg/mL in deionized water, DMEM-H pH 7.4, DMEM-H with 7 mM lactate pH 6.5, or DMEM-H 10% FBS pH 7.4, vortexed, and centrifuged for 10 min at 15,000 ×g, room temperature, to clear insoluble particles. Rhodamine B was also dissolved at 1 µg/mL in the same media conditions. Media fluorescence was measured against a standard curve of 0.2 to 2 µg/mL rhodamine B (to ensure reproducibility between plates), in triplicate wells of black FluoNunc 96-well plates with a Molecular Dynamics Gemini II fluorescent plate reader (Sunnyvale, CA, USA) set at 550 nm excitation, 580 nm emission, 570 nm cutoff. RITC-chitosan media solubility was calculated as the ratio of the fluorescence of cleared media supernatant divided by the fluorescence in water. Results were calculated as the mean and standard deviation from three independent sample preparations.

### 3.5. Flow Cytometry

HEK293 cells were seeded at three million cells per well in 6-well plates or 35 mm Petri dishes and cultured as confluent monolayers overnight in DMEM with 10% FBS. Confluent cells were used, to ensure that all chitosan particles settled in contact with cells and not with the surface of the plastic tissue culture petri. Cells were rinsed with pre-warmed DMEM and overlaid for 4 h with 2.5 mL pre-warmed DMEM or DMEM with 10% FBS, or for 24 h with DMEM containing 0%, 2%, 5% or 10% FBS, or 10% heat-inactivated serum. Heat-inactivated serum was generated by 30 minutes incubation at 56 °C. Control wells received no chitosan (negative control), or 1 µg/mL rhodamine B. Treated wells received 25 µL of filter-sterile 5 mg/mL RITC-chitosan (in dilute HCl, pH 5.6), pipetted directly into 2.5 mL media and the petri agitated briefly in a cruciform fashion to distribute chitosan particles evenly over the cells at a 50 µg/mL final concentration. After 4 or 24 h of incubation in a humidified incubator at 37 °C, 5% CO_2_, cells were labeled for 5 min with 2 µg/mL Hoescht 33342, rinsed with PBS, treated for 5 to 10 min with trypsin-EDTA, centrifuged in DMEM+10% FBS, resuspended in DMEM or PBS and kept on ice until analyzed using a MoFlo Cytometer (Cytomation, Denver, CO, USA). For different uptake experiments, the FL4 channel (530 nm excitation at 200 mW, emission filter 580/60) was adjusted to have an appropriate dynamic range. Serum-opsonized chitosan particles were generated by incubating chitosan for 1 h at 37 °C in DMEM with 10% FBS, followed by centrifugation to collect chitosan particles, washing in DMEM pH 7.4 to remove residual serum, and resuspended in DMEM at the same 50 µg/mL concentration to apply to cells. Unlabeled cells, double-positive red (RITC-chitosan) and blue (Hoechst-stained nuclei) cells, and pulse-width gated events were used to exclude cell debris, cell doublets, and free RITC-chitosan particles from the analyses. The linear relative fluorescence (1 to 256) was converted to log scale values (from 1 to 10,000) using the equation Log X = 10*X/64 in order to generate the graph profiles for Figure 7 and Figure 9, using Microsoft Excel. The MoFlo software automatically reported the data as mean fluorescence, and percent gated cells labeled. All data were reproducible (N = 3 to N = 4 independent cultures on separate occasions, except for lactate+DMEM, N = 2 separate occasions and serum-opsonized chitosan particles, N = 1 experiment with two distinct chitosans).

To generate cell cytospins, cell suspensions prepared for flow cytometry (at approximately 10^6^ cells per mL) were diluted by pipetting 100 µL cells into 1.5 mL room temperature phosphate buffered saline. Glass microscope slides cleaned with 70% ethanol to remove dust particles were placed in cytospin holders with a single funnel and a filter paper adapter. 500 µL of diluted cells were pipetted into the funnel, and the cytospin (Rotofix 23, Hettich Lab Technology, Tuttlingen, Germany) centrifuged for 5 minutes at 500 rpm. Cells were allowed to air dry then imaged with an inverted epifluorescent microscope equipped with a digital camera and calibrated histomorphometry software (Northern Eclipe, Empix, Missisauga, ON, Canada) by collecting red fluorescent, blue fluorescent and low-intensity brightfield images of the same field at 20x magnification that were merged into one digital image.

### 3.6. Live Confocal Microscopy

Confluent monolayer HEK293 cells were cultured in DMEM or DMEM+10% FBS initial pH 7.4, with or without 50 µg/mL RITC-chitosan (80M, 95M, 80L, 95L) or 1 µg/mL rhodamine B for 24 h. Uptake of RITC-80M after 24 h was also analyzed in DMEM pH 7.4, or DMEM supplemented with 7 mM lactate (media pH ~6.5), or DMEM pH 7.4 with EGF (30 ng/mL or 100 ng/mL, to promote macropinocytosis [45]), or DMEM with 10% FBS pH 7.4. After RITC-chitosan incubation, cells were further labeled with 1 µg/mL calcein AM (30 min incubation, 37 °C, vital fluorescent green cytosolic stain), then with 2 µg/mL Hoechst 33342 (5 min incubation, 37 °C, fluorescent blue nuclei) and then rinsed and placed in DMEM for confocal imaging using a Carl Zeiss LSM 510 META Axioplan 2 confocal scanning microscope equipped with a Plan-Apochromat 63x/1.0NA water-immersion objective (Carl Zeiss AG, Feldbach, Switzerland). 3D images were produced using the software Imaris (Bitplane, Zurich, Switzerland).

### 3.7. Lactate and Glucose Measurements

The YSI 2700 SELECT Biochemistry analyzer (Life Sciences, Yellow Springs, OH, USA) was used to determine the concentration of L-lactate and D-glucose in unconditioned and 24-hour conditioned cell culture media in cells cultured with or without serum, or EGF, or lactate-acidified media, and exposed or not to RITC-chitosan 80M. Data were analyzed as the mean level of L-lactate produced (*i.e.*, final conditioned media L-lactate concentration subtracted for initial media L-lactate concentration) and depletion of D-glucose (initial D-glucose subtracted for final D-glucose) obtained from independent cell samples generated during 2 or 3 distinct cultures, with N = 3 to 5 (DMEM, DMEM+2% to 10% FBS, lactate, and EGF) and data shown as the mean ± SD.

### 3.8. Statistical Analysis

The Student’s t-test was used to test the effect of serum and DDA on RITC-chitosan fluorescence in media cleared on insoluble precipitates (N = 3 to 5 distinct samples per chitosan and two chitosan types per DDA), the effect of cells and serum on media pH (N = 3 to 6 readings from cultures carried out on separate occasions), rhodamine B fluorescence in culture media *vs.* deionized water (N = 3) and zeta potential of RITC-chitosan in DMEM+10% serum *vs.* DMEM+10% serum. One-way analysis of the variance of the mean (ANOVA) was used to analyze the effect of media condition on mean FL4 channel (red excitation/emission) cell fluorescence and percent cells labeled, in a population of 5,000 to 20,000 cells analyzed by flow cytometry, using Statistica software (StatSoft, version 6.1, Tulsa, OK, USA). Significance was set at *p* < 0.05.

## 4. Conclusions

The chitosans analyzed in this study are acid-soluble polysaccharides that form insoluble neutral microparticles when added to standard cell culture media (pH 7–7.5). In serum-containing media pH 7.4, non-biodegradable chitosan was 99% insoluble, and was internalized more rapidly than 80% DDA chitosan after 4 h of incubation. The delay in cell uptake of 80% DDA chitosan *in vitro* can be explained by the slight but significantly enhanced solubility of 80%DDA chitosan in the presence of serum, compared to 95% DDA chitosan, which delayed particle precipitation onto the cell monolayers. The dose-dependent effect of serum on chitosan particle uptake, and observed media acidification specifically in the presence of serum, suggest that serum up-regulates cell metabolism required for macropinocytosis [48]. By contrast, in serum-free medium pH 6.5, a significant 30% to 50% of the chitosan remains soluble and becomes taken up at low-levels in small intracellular vesicles under conditions of relatively low metabolism (lactated DMEM). These data suggest that soluble cationic chitosan chains can enter the cell through non-specific fluid-phase pinocytosis. This study has clarified the role of serum and media pH on chitosan solubility and cell internalization. Soluble chitosan chains can enter the cell through fluid-phase pinocytosis while serum-biofouled chitosan particles can only be engulfed through high energy-dependent processes stimulated by serum.

## Figures and Tables

**Table 1:** Insoluble chitosan microparticles: Published examples of *in vivo* biomedical use, and *in vitro* investigation of cell responses.

Method of insoluble chitosan microparticle preparation	Chitosans analyzed	Application
***Neutral precipitation of acid-soluble chitosan *in vivo* or *in vitro****		
Mix acid-soluble chitosan solution or chitosan-glycerol phosphate solution pH 4–6.8 with whole blood and/or directly apply to bleeding tissues	80% DDA 75%–82% DDA, 150–250 kDa	Hemostatic *in vivo* [17] Bone and articular cartilage repair *in vivo* [20]
Inject mixtures of chitosan-DNA, with a molar excess of chitosan, in intramuscular, subcutaneous sites	92% DDA, 10 kDa 80% DDA, 10 kDa 80% DDA, 80 kDa	Gene delivery *in vivo* [24]
Combine soluble chitosan at 5- to 10-fold molar excess with DNA, pipette into cell culture medium DMEM+10% serum pH 7.6	80% DDA, 15 kDa 92% DDA, >100 kDa 94% DDA, 52 kDa	*In vitro* DNA delivery: A549, Hela, B16 cells, HEK293 cells [25,26]
Pipette acid-soluble chitosan pH 5.0 into basal media pH 7.6 (DMEM, αMEM, RPMI±10% to 16% fetal bovine serum)	80% DDA, 179 kDa 81% DDA, 35 kDa 80% DDA, 179 kDa 95% DDA, 168 kDa 92% DDA, 10 kDa	*In vitro* bone marrow stromal cell osteogenesis [27] *In vitro* macrophage activation [28] *In vitro* neutrophil chemotaxis, degranulation, chitosan uptake [14] *In vitro* chitosan-HEK293 cell adsorption/uptake [26]
***Pre-formed chitosan microparticles***		
Pre-formed microparticles (glutaraldehyde and Tween surfactant) injected into the mouse foot pad or added to DMEM+heat-inactivated 10% fetal bovine serum	75%–85% DDA, 164 kDa	Vaccine, lymph node trafficking *in vivo* [29] *In vitro* HEK293, A549, RAW264 cell uptake [29]
1 µm or 3.5 µm pre-formed chitosan microparticles added to RPMI+10% serum, pH 7.2	≥80% DDA	Wound-repair applications: *in vitro* neutrophil chemotaxis [30] *in vitro* macrophage activation [31]

**Table 2:** RITC-chitosans used in the study.

*chitosan*	*Viscosity (mPa.s) ^*	*Chitosan DDA (%) #*	*M_n_ (kDa)*	*PDI (M_w_/M_n_)*	*RITC/chitosan % mol/mol*
RITC-95M *	2,964	94.6	177	1.1	0.6
RITC-95L *	197	94.6	102	1.2	0.6
RITC-80M *	1,422	80.6	144	1.3	0.5
RITC-80L *	178	80.2	108	1.5	0.5
RITC-82M **	N.D.	81.7	241	1.1	0.9

^ 2.05% w/w HCl solution pH 5.6 and # %DDA prior to RITC-labeling *Mn:* number-average molecular weight. PDI: polydispersity index; N.D.: not done; * used for particle size, chitosan solubility, cell uptake; ** used for FT-IR (Figure 3B).
